# Alexithymia and Problematic Mobile Phone Use: A Moderated Mediation Model

**DOI:** 10.3389/fpsyg.2020.541507

**Published:** 2020-09-15

**Authors:** Zejun Hao, Liangyi Jin

**Affiliations:** ^1^School of Fundamental Sciences, China Medical University, Shenyang, China; ^2^Shenyang Women’s and Children’s Hospital, Shenyang, China

**Keywords:** alexithymia, problematic mobile phone use, moderated mediation model, mindfulness facets, Chinese undergraduate students

## Abstract

Alexithymia has been studied with its impact on problematic mobile phone use. However, none of these studies has examined the roles of mindfulness facets in this relationship. To address this issue, a total of 901 Chinese undergraduate students participated in this study and were tested with questionnaires for measuring their levels of alexithymia, problematic mobile phone use, and mindfulness facets. A moderated mediation model was constructed to examine the roles of mindfulness facets in the association between alexithymia and problematic mobile phone use. The results showed that after controlling for age and sex, alexithymia positively predicted problematic mobile phone use both directly (β = 0.157, *p* < 0.001) and indirectly via mindfulness facet of acting with awareness (16^th^, 50^th^, and 84^th^ percentiles of observing were −5.371, β = 0.019; −0.371, β = 0.216; and 4.629, β = 0.242, respectively, and the 95% confidence intervals were 0.142 to 0.246; 0.167 to 0.269; and 0.186 to 0.3, respectively). The facet of acting with awareness partially mediated this relationship in a negative way. Further, the facet of observing moderated the path between alexithymia and the facet of acting with awareness: with a higher level of observing, the negative association between alexithymia and acting with awareness became more negative (alexithymia × observing, β = −0.006, *p* = 0.001, 95% confidence interval −0.01 to −0.003). The current study advanced our understanding of the mechanism underlying the connection between alexithymia and problematic mobile phone use and helped to investigate how mindfulness skills benefited the individuals.

## Introduction

Modern devices such as the multifunctional mobile phones have reshaped people’s ways of communication and their lifestyles. In China, the number of people who access the internet via mobile phones has gone up to 897 million as of March 2020 (The 45th China Statistical Report on Internet Development, 2020). Mobile phone use, which has become an integral part of daily life, brings the benefits of convenience and somehow increasing productivity. Meanwhile, several studies had also investigated different behavioral and mental health problems because of mobile phone overuses. Alexithymia has been observed as a robust predictor of problematic mobile phone use (Gao et al., 2018; Mei et al., 2018; Hao et al., 2019). However, none of the studies examined the roles of mindfulness facets in this relationship, although mindfulness facets have displayed good effects for promoting psychological well-being.

### Problematic Mobile Phone Use

This study concentrates on the disordered mobile phone use. The literature study elucidates that mobile phone jumbling has two synonyms: “mobile phone addiction” and “problematic mobile phone use” (Hwang and Park, 2015; Enez Darcin et al., 2016; Jiang and Zhao, 2016; Liu et al., 2017). Given that there has been no consensus over applying the term “addiction” to mobile phone use and that the disordered mobile phone use has not been listed in both the Diagnostic and Statistical Manual of Mental Disorders, Fifth Edition, and the International Classification of Disease, 11^th^ Revision, we choose the term “problematic mobile phone use” in this study. Despite some scholars proposed the similar features of problematic mobile phone use and some recognized behavioral addictions, there have been to date no formal diagnostic criteria for problematic use of the mobile phone. Billieux (2012) defined problematic mobile phone use as an inability to regulate one’s use of the mobile phone, which has negative consequences in daily life. Previous studies have documented the detrimental impacts of interfered sleep quality (Hisli Sahin et al., 2009; Tamura et al., 2017), physical impairments (AlAbdulwahab et al., 2017), deficit interpersonal capabilities (Chen et al., 2016), and mental health issues (Thomée et al., 2011). Young people are active users of mobile phones and are very likely afflicted by problematic uses. Therefore, the issue of problematic mobile phone use in the student population has got attention in mental health research. The estimated prevalence rates are 10–30% among adolescents and 10–46% among college students (Jun, 2016; Lian et al., 2016). The consequences of problematic mobile phone use are appearing among students where they are distracted from their academic learnings (Ravizza et al., 2014) and show a decline in their academic performances (Çağan et al., 2014; Lepp et al., 2014; Liu and Yoo, 2018). The predisposing factors of problematic mobile phone use have also been examined. Augner and Hacker (2012) described that personality traits of extroversion and neuroticism were positively correlated with problematic mobile phone use. Andreassen et al. (2013) found that openness, agreeableness, and conscientiousness displayed negative associations with problematic mobile phone use. Furthermore, low self-control, low self-esteem, perceived stress, and high levels of anxiety and depression had been found to positively predict problematic mobile phone use (Bianchi and Phillips, 2005; Jiang and Zhao, 2016; Liu et al., 2018; Elhai et al., 2020).

### Alexithymia

Alexithymia refers to the difficulties in identifying and describing emotions in self and others, restricted imagination, and the externally oriented way of thinking (Taylor et al., 1997). Alexithymia has been treated as a personality trait (Salminen et al., 2006; de Timary et al., 2008) and is linked to impairments in physical (Pollatos and Gramann, 2012; Khosravani et al., 2016) and mental health. A study had reported that approximately 36% of Chinese adolescents are affected by alexithymia (Gao et al., 2018). Mattila et al. (2010) had suggested that the core features of alexithymia are the inadequate abilities to sense and respond to the feelings in self and others, which ultimately increase the chances to develop interpersonal problems in an individual. These interpersonal problems bring troubles in building and maintaining social circles of a person (Timoney and Holder, 2013). When situated in unfavorable conditions, individuals with alexithymia gain less social support (Mallinckrodt and Wei, 2005) and are less competent to cope with stressful challenges (Hisli Sahin et al., 2009). In addition, due to the deficiencies in emotion regulation (Taylor et al., 1997; Ghorbani et al., 2017), negative feelings of depression and anxiety are more likely to be observed on those with a high level of alexithymia (Timoney and Holder, 2013; Nekouei et al., 2014).

### Alexithymia and Problematic Mobile Phone Use

Individuals with alexithymia tend to use addictive behaviors to escape from the challenges of real-life (Schimmenti et al., 2017). Avoiding real-life problems is an emerging behavior among young mobile phone users (Chiu, 2014; Wang et al., 2015). The developing scenario of young mobile phone users highlights the necessity to investigate the association between alexithymia and problematic mobile phone use. A previous study had reported that the scores of alexithymia of individuals with problematic mobile phone use are significantly higher than those of the control group (Ha et al., 2008). Recent developments in this field of research proved some overviews that alexithymia was a key predisposing factor of problematic mobile phone use (Mei et al., 2018; Hao et al., 2019). Alexithymia is linked with negative emotions of depression and anxiety (Nekouei et al., 2014), and to get rid of these emotional disorders, individuals with alexithymia would turn to mobile phones for the instant gratification (Gao et al., 2018; Hao et al., 2019). The deficit cognitive function brings frustrations when individuals with alexithymia seek to build and maintain friendships via face-to-face interactions (Jordan and Smith, 2017). Hence, online communications through mobile phones may become their first option. This choice of the virtual world leads individuals to avoid physical contact and proximity to others. This condition ultimately enhances the likelihood of developing problematic mobile phone use.

### Mindfulness

Mindfulness is rooted in Buddhism and has been described as purposefully paying attention to the present moment in a non-judgmental way (Kabat-Zinn, 1994; Baer et al., 2006). Mindfulness could promote psychological well-being and has been applied in clinical trials for reducing the levels of pain (Kabat-Zinn, 1990), depressive symptoms (Segal et al., 2002), and substance use (Witkiewitz et al., 2005). Shapiro et al. (2006) proposed the three core components of mindfulness: attention, intention, and attitude. There is an argument that a shifted perspective (naming reperceiving) is cultivated through mindfulness practice (Shapiro, 2009). It is a meta-mechanism or a mechanism that mobilizes other mechanisms for promoting psychological outcomes. In a study, researchers reported that after receiving the mindfulness-based intervention, participants showed a significantly lower level of alexithymia (Santarnecchi et al., 2014). In addition, it had been found that if we enhance an individual’s level of awareness, the mindfulness displays a protective role for problematic habitual behaviors such as disordered mobile phone use (Lan et al., 2018). Li and Hao (2019) have reported the moderation effect of mindfulness in the positive relationship between alexithymia and problematic mobile phone use where the adverse impact was weakened under the condition of high level of mindfulness. However, there is no study to our knowledge that has examined the specific roles of the mindfulness facets in this relationship. We believe this research would help to investigate how mindfulness skills benefit the individuals.

#### Mindfulness Facets as Mediators

In this study, we have used the Five-Facet Mindfulness Questionnaire (FFMQ; Baer et al., 2006) to assess the mindfulness facets: (1) observing (*noticing internal and external experiences*), (2) describing (*describing the inner feelings*), (3) acting with awareness (*attending to the moment*), (4) non-judging of experience (*being able to accept the feelings, avoid judging one’s own experience*), and (5) non-reactivity to inner experience (*being able to notice feelings and let them go and come without reacting to them*). Although these five subscales belong to one questionnaire, many studies have indicated that some facets showed negative correlations with each other (de Bruin et al., 2012; Boden et al., 2015; Parsons et al., 2017; Haas and Akamatsu, 2019; Single et al., 2019). This could be explained by that instead of being used as a unified scale, FFMQ was originally conceptualized to treat mindfulness as multidimensional. Moreover, it has been used to measure the five independent aspects of mindfulness with good validities (Baer et al., 2006; Brown et al., 2015; Haas and Akamatsu, 2019). In addition, when FFMQ is applied in a non-meditating sample, the results obtained may be different from those with meditating experiences (de Bruin et al., 2012). In previous studies on the mindfulness facets and alexithymia, alexithymia was negatively associated with all the five facets of mindfulness (de Bruin et al., 2012; MacDonald and Price, 2017). However, there is a study on mindfulness facets and problematic mobile phone use that reported mixed findings. That investigation depicted that mindfulness facets of describing, acting with awareness, and non-judging of inner experience were negatively associated with problematic mobile phone use, whereas observing and non-reactivity to inner experience displayed positive correlations with problematic mobile phone use (Owen et al., 2018). Altogether, we hypothesized that alexithymia would positively affect problematic mobile phone use and all the mindfulness facets would significantly mediate this relationship where observing and non-reactivity to inner experience would mediate this association in positive ways and describing, acting with awareness, and non-judging of inner experience would mediate this association in negative ways.

#### Mindfulness Facet of Observing as a Moderator

The “observing” subscale of FFMQ has been used to measure the level of attention to the internal and external experiences (Carmody et al., 2009). In mindfulness practice, paying attention includes observing the present moment, internal and external experiences. Observing has been suggested as a critical role in the healing process, and mindfulness-based cognitive therapy is based on the capacity to observe internal and external behaviors (Segal et al., 2002). Observing is the prerequisite of mindfulness practice because without being properly aware of the stimuli, the other facets of describing, acting with awareness in a non-judgmental way, would not be intrigued. In addition, the ability to observe would compensate for the deficit skills of alexithymics to identify and describe the emotions. Therefore, we hypothesized that with higher observing, the relationship between alexithymia and mindfulness facets would become less negative.

#### Moderated Mediation Effects

The moderated mediation effects refer to the conditional indirect effects or the magnitude of an indirect effect at a particular value of a moderator (or at particular values of more than one moderator) (Preacher et al., 2007). Among the proposed methods and procedures, the conditional PROCESS analysis, which is an SPSS macro provided by Preacher et al. (2007), had been widely used in a large number of studies (Liu et al., 2018; Nam et al., 2018; Hao et al., 2019). In the current study, if the mediation effects of mindfulness facets coexisted with the moderation effect of observing on the indirect paths, then the moderated mediation effects existed between alexithymia and problematic mobile phone use.

### Current Study

**FIGURE 1:**
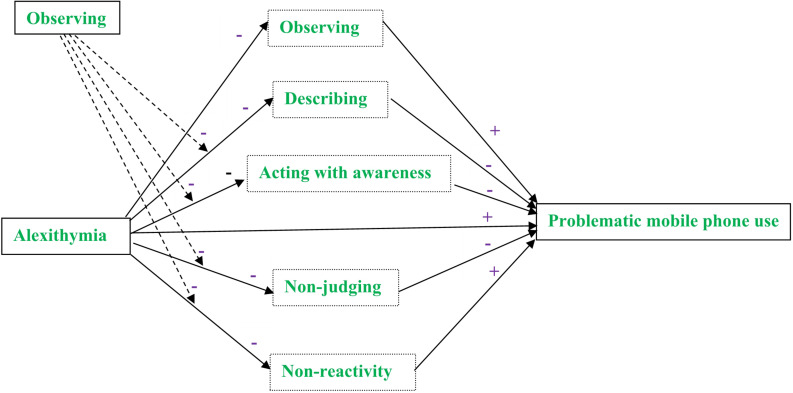
The hypothesized model. The dotted boxes contained the investigated mediators and the dotted lines indicated the investigated moderation effects.

The current study constructed a moderated mediation model to explore the mediation and the moderation effects of mindfulness facets in the association between alexithymia and problematic mobile phone use. The objectives of the current study were twofold. Firstly, we aimed to examine the mediating roles of mindfulness facets in the relationship between alexithymia and problematic mobile phone use. We hypothesized that all the mindfulness facets would significantly mediate this positive association, where observing and non-reactivity to inner experience would positively mediate this association and describing, acting with awareness, and non-judging of inner experience would negatively mediate this association. Secondly, the current study explored the potential protective role of observing for alexithymia. We hypothesized that with higher observing levels, the negative impacts of alexithymia on the mediating mindfulness facets would become less negative. The hypothesized model is depicted in Figure 1.

## Materials and Methods

### Participants and Procedure

The survey was conducted in Liaoning Province, Northeast China. To guarantee the diversity of participants, the two universities selected were (1) Liaoning National Normal College, where the students get enrolled in different disciplines including Education of Chinese language, English, Art and Music, etc. and (2) Shenyang University of Technology. The stratified cluster sampling method was used. The participants were undergraduate students (from grades 1 to 4), and two classes of students randomly selected from each grade were invited to take part in the survey. Electronic questionnaires were forwarded to the participants via the WeChat and QQ groups. It took 30 min to complete all the questions. Answers were collected automatically when participants clicked the submit button. Written consent was obtained from every participant. A total of 998 students responded, and 901 were valid for analyses (97 responded with missing data). The participants were between 18 and 24 years of age, with an average age of 20.132 years. There were 473 male students (52.5%), and 428 (47.5%) were female participants. The Ethical Committee of Liaoning National Normal College approved the survey. A small gift of the key chain was presented to each participant as appreciation and encouragement for participating in the survey.

### Measures

#### Problematic Mobile Phone Use

The self-report Mobile Phone Addiction Index was used to measure the tendencies of problematic mobile phone use among participants (Leung, 2008). The questionnaire has been proved with good validity for the Chinese population (Gao et al., 2018). It involves 17 items, runs on a five-point Likert scale system, and ranges from “does not describe me well” (1) to “describes me very well” (5). The higher score indicates the higher tendency of problematic mobile phone use. Sample items include, e.g., “Your friends and family complained about your mobile phone usage” and “You have been told that you spend too much time on your mobile phone.” The Cronbach’s alpha in this research was 0.883.

#### Alexithymia

The Toronto Alexithymia Scale-20 (Bagby et al., 1994) was used to measure an individual’s alexithymia level. It includes 20 items and is based on a five-point Likert system ranging from “does not describe me well” (1) to “describes me very well” (5). It has been proved with good validity among Chinese participants (Han et al., 2012; Zhang et al., 2017). Sample items include, e.g., “I am often confused about what emotion I am feeling” and “It is difficult for me to find the right words for my feelings.” The higher score indicates a higher level of alexithymia. The Cronbach’s alpha in this research was 0.807.

#### Mindfulness Facets

The Five Facet Mindfulness Questionnaire (Baer et al., 2006) was used to measure the mindfulness facets of observing, describing, acting with awareness, non-judging of experience, and non-reactivity to inner experience. It includes 39 items and runs on a five-point Likert scale system ranging from “never or very rarely true” (1) to “very often or always true” (5). The higher scores indicated the higher levels of each tested facet. Sample items include, e.g., “When I’m walking, I deliberately notice the sensations of my body moving” (*observing*); “I’m good at finding words to describe my feelings” (*describing*); “When I do things, my mind wanders off and I’m easily distracted (reversed item)” (*acting with awareness*); “I criticize myself for having irrational or inappropriate emotions (reversed item)” (*non-judging of experience*); and “I perceive my feelings and emotions without having to react to them” (*non-reactivity to inner experience*). The Cronbach’s alpha of each subscale was observing = 0.796, describing = 0.630, acting with awareness = 0.852, non-judging of experience = 0.778, and non-reactivity to inner experience = 0.734 indicating all facets had high reliabilities.

#### Data Analyses

SPSS 22.0 was used for statistical analyses in this study. The significant level was set at 0.05. First, we checked the distribution of the data and found they were in non-normal distribution. According to Preacher et al. (2007) and Hayes (2015), the analyses via the SPSS PROCESS macro had no requirement on the distribution of the data. In this study, Spearman’s analysis was conducted to examine the correlations of all the investigated variables. Second, model 4 of SPSS macro PROCESS 3.1 (Hayes, 2018) was used to test the mediating roles of mindfulness facets in the relationship between alexithymia and problematic mobile phone use by generating bias-corrected bootstrap confidence interval (using 5,000 bootstrapping samples), and third, the moderated mediation model was tested with model 7 by using the same method and software as described for model 4. Considering the difference of age and sex in alexithymia and problematic mobile phone use (Chen and Chung, 2016; De-Sola Gutiérrez et al., 2016), age and sex were treated as controlled variables in the present study.

## Results

### Preliminary Analyses

Spearman’s analysis indicated that all the investigated variables had significant correlations with each other. Alexithymia was significantly positively associated with problematic mobile phone use. The relationship between alexithymia and mindfulness facets yielded mixed results. Alexithymia was in negative associations with mindfulness facets of describing, acting with awareness, and non-judging of experience but in positive associations with observing and non-reactivity to inner experience (see Table 1).

**TABLE 1:** Spearman correlation coefficients of all the investigated variables.

	**1**	**2**	**3**	**4**	**5**	**6**	**7**
(1) Alexithymia							
(2) Observing	0.243***						
(3) Describing	−0.275***	0.209***					
(4) Acting with awareness	−0.551***	−0.478***	0.068*				
(5) Non-judging of experience	−0.269***	−0.616***	−0.145***	0.544***			
(6) Non-reactivity to inner experience	0.282***	0.575***	0.186***	−0.503***	−0.609***		
(7) PMU	0.340***	0.208***	−0.053***	−0.439***	−0.281***	0.202***	
Median	57.000	22.000	24.000	28.000	25.000	19.000	41.000
P_25_, P_75_	50,62	19,26	22,26	24,32	22,28	16,21	33,49

*N* = 901, * *p <* 0.05; ^∗∗∗^*p* < 0.001. PMU, problematic mobile phone use. P_*25*_ = 25^*th*^ percentile, P_*75*_ = 75^*th*^ percentile. The data were in non-normal distribution and Spearman analysis was conducted accordingly.

### Testing for the Mediation Effects of Mindfulness Facets

The results of the mediation analyses showed that after controlling for age and sex, alexithymia positively predicted problematic mobile phone use both directly (β = 0.189, *p* < 0.001) and indirectly via mindfulness facet of acting with awareness (β = 0.242, 95% confidence interval 0.175 to 0.308) (see Tables 2, 3). Namely, the acting with awareness facet of mindfulness partially mediated the impact of alexithymia on problematic mobile phone use in a negative way. There was no significant mediating effects observed for the other four detected paths (observing, β = 0.000, 95% confidence interval −0.025 to 0.023; describing, β = −0.021, 95% confidence interval −0.051 to 0.007; non-judging of experience, β = 0.025, 95% confidence interval −0.005 to 0.058; non-reactivity to inner experience, β = −0.015, 95% confidence interval −0.047 to 0.016).

**TABLE 2:** Mediation analysis.

**Outcome variable**	**Independent variables**	**β**	***SE***	***t***	***p***
PMU	Constant	32.397***	6.425	5.043	<0.001
	Age	−0.807**	0.296	–2.729	<0.01
	Gender ^a^	1.454*	0.734	1.982	<0.05
	Alexithymia	0.419***	0.037	11.443	<0.001
Observing	Constant	20.280***	3.146	6.446	<0.001
	Age	–0.194	0.145	–1.340	0.180
	Gender ^a^	−0.836*	0.359	–2.326	<0.05
	Alexithymia	0.130***	0.018	7.228	<0.001
Describing	Constant	31.393***	2.252	13.940	<0.001
	Age	–0.010	0.104	–0.099	0.922
	Gender ^a^	0.469	0.257	1.823	0.069
	Alexithymia	−0.144***	0.013	–11.223	<0.001
Acting with awareness	Constant	38.261***	2.684	14.253	<0.001
	Age	0.286*	0.124	2.313	<0.05
	Gender ^a^	0.947**	0.307	3.091	<0.01
	Alexithymia	−0.319***	0.015	–20.836	<0.001
Non-judging of experience	Constant	23.735***	2.853	8.320	<0.001
	Age	0.459***	0.131	3.494	<0.001
	Gender ^a^	0.032	0.326	0.099	0.921
	Alexithymia	−0.144***	0.016	–8.846	<0.001
Non-reactivity to inner experience	Constant	16.337***	2.360	6.923	<0.001
	Age	–0.145	0.109	–1.331	0.184
	Gender ^a^	−0.856**	0.269	–3.176	<0.01
	Alexithymia	0.120***	0.013	8.931	<0.001
PMU	Constant	62.932***	8.113	7.757	<0.001
	Age	–0.528	0.280	–1.887	0.059
	Gender ^a^	2.001*	0.702	2.850	<0.05
	Observing	–0.003	0.089	–0.031	0.975
	Describing	0.148	0.096	1.538	0.124
	Acting with awareness	−0.759***	0.092	–8.259	<0.001
	Non-judging of experience	–0.172	0.103	–1.662	0.097
	Non-reactivity to inner experience	–0.124	0.115	–1.072	0.284
	Alexithymia	0.189***	0.045	4.226	<0.001

*N* = 901, * *p* < 0.05, ** *p* < 0.01, *** *p* < 0.001. PMU, problematic mobile phone use. Gender^*a*^, Male = 1, female = 2.

**TABLE 3:** Bootstrapping indirect effect and 95% confidence interval (CI) for the mediation test.

**Indirect path**	**Estimated effect**	**95% CI**	
		**BootLLCI**	**BootULCI**
Alexithymia → observing → PMU	0.000^b^	–0.025	0.023
Alexithymia → describing → PMU	−0.021^b^	–0.051	0.007
Alexithymia → acting with awareness → PMU	0.242^a^	0.175	0.308
Alexithymia → non-judging of experience → PMU	0.025^b^	–0.005	0.058
Alexithymia → non-reactivity to inner experience → PMU	−0.015^b^	–0.047	0.016

**N* = 901. Bootstrap sample size = 5000. PMU, problematic mobile phone use; LL, low limit; CI, confidence interval; UL, upper limit. ^*a*^Empirical 95% confidence interval does not overlap with zero. ^*b*^Empirical 95% confidence interval overlaps with zero.*

### Testing for the Moderated Mediation Effects

**FIGURE 2:**
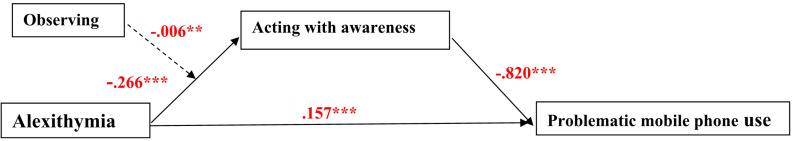
The verified model of the moderated mediation effects between alexithymia and problematic mobile phone use (*N* = 901). The study found that acting with awareness negatively mediated the association between alexithymia and problematic mobile phone use. Observing significantly moderated the impact of alexithymia on acting with awareness. **p < 0.01, ***p < 0.001.

**FIGURE 3:**
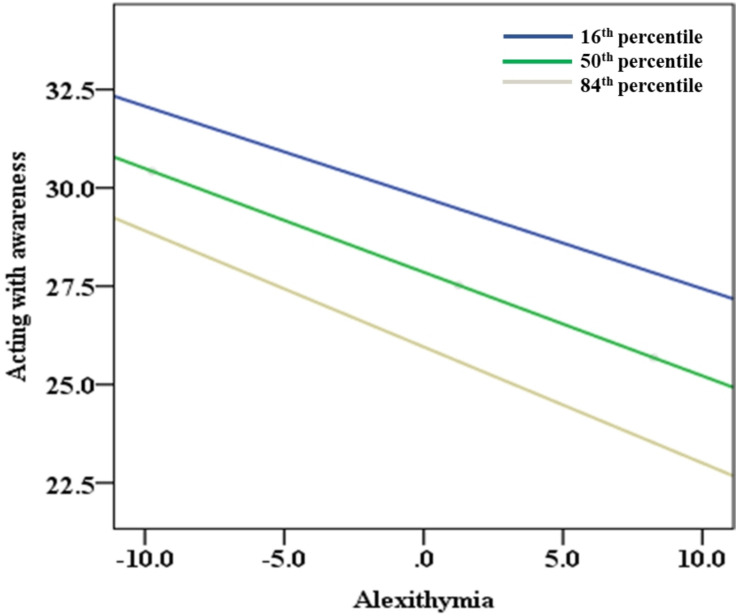
The conditional effect between alexithymia and acting with awareness (*N* = 901). To further understand the moderation effect of observing on the path between alexithymia and acting with awareness, a conditional indirect effect analysis was conducted at 16^th^, 50^th^, and 84^th^ percentiles of the observing facet.

**FIGURE 4:**
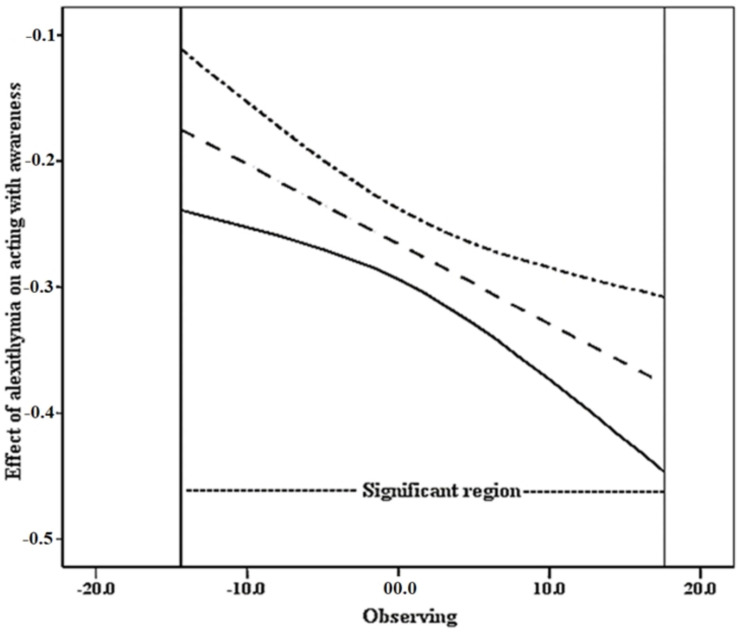
Effects of observing with 95% confidence interval (*N* = 901). The Johnson-Neyman (J-N) method was applied to measure the significant region in which observing significantly moderated the impact of alexithymia on acting with awareness. The upper and lower lines indicated the limits of confidence interval and the middle line indicated the effect of observing. The scores of statistical significance ranged from –14.371 to 17.629.

The results of the test for the moderated mediation effects by SPSS macro PROCESS 3.1 (Hayes, 2018) are shown in Tables 4, 5. After controlling for age and sex, alexithymia positively predicted problematic mobile phone use both directly (β = 0.157, *p* < 0.001) and indirectly via the facet of acting with awareness (16^th^ percentile of observing = −5.371, β = 0.019, 95% confidence interval 0.142 to 0.246; 50^th^ percentile of observing = −0.371, β = 0.216, 95% confidence interval 0.167 to 0.269; 84^th^ percentile of observing = 4.629, β = 0.242, 95% confidence interval 0.186 to 0.3). Namely, the facet of acting with awareness partially mediated the relationship between alexithymia and problematic mobile phone use in a negative way. The interaction of alexithymia and observing had a significant effect on acting with awareness: with a higher level of observing, the negative association between alexithymia and acting with awareness became more negative (alexithymia × observing, β = −0.006, *p* = 0.001, 95% confidence interval −0.01 to −0.003). Namely, observing moderated the impact of alexithymia on acting with awareness. The moderated mediation model proposed in this study was supported, according to Hayes (2015). The verified moderated mediation model is depicted in Figure 2. For the further understanding of the moderation effect of observing on the path between alexithymia and acting with awareness, a conditional indirect effect analysis was conducted at 16^th^, 50^th^, and 84^th^ percentiles of the observing facet (see Table 4 and Figure 3). Statistically significant region or the range of scores in which observing moderated, the Johnson–Neyman method was applied for a better presentation (see Figure 4). The analysis showed that the scores of statistical significance ranged from −14.371 to 17.629. Table 5 showed the conditional indirect effect between alexithymia and problematic mobile phone use. Among individuals with low observing levels, the indirect impact of alexithymia on problematic mobile phone use was weak, whereas among individuals with high observing levels, the indirect impact would become stronger.

**TABLE 4:** The moderated mediation analyses.

**Outcome variable**		**Independent variable**	**β**	***SE***	***t***	***p***	**BootLLCI**	**BootULCI**
Acting with awareness		Constant	22.655***	2.310	9.807	0.000	18.121	27.189
		Age	0.210	0.111	1.887	0.060	–0.008	0.428
		Gender ^a^	0.568*	0.277	2.050	0.041	0.024	1.112
		Observing	−0.380***	0.026	–14.673	0.000	–0.431	–0.329
		Alexithymia	−0.266***	0.014	–18.672	0.000	–0.294	–0.238
		Alexithymia x observing	−0.006**	0.002	–3.267	0.001	–0.010	–0.003
PMU		Constant	72.552***	5.973	12.146	0.000	60.829	84.276
		Age	−0.573*	0.279	–2.054	0.040	–1.120	–0.025
		Gender ^a^	2.231**	0.693	3.217	0.001	0.870	3.591
		Acting with awareness	−0.820***	0.075	–10.909	0.000	–0.967	–0.672
		Alexithymia	0.157***	0.042	3.762	0.000	0.075	0.240

**Conditional indirect effect analysis at the values of the moderator**		**β**		***SE***	***t***	**BootLLCI**		**BootULCI**
**%**	**Moderator: observing (mean centered)**							

16^th^	−5.371	−0.232		0.019^b^	–12.490	−0.268		–0.196
50^th^	−0.371	−0.264		0.014^b^	–18.378	−0.292		–0.235
84^th^	4.629	−0.295		0.016^b^	–18.542	−0.326		–0.264

*N* = 901, * *p* < 0.05, ** *p*< 0.01, *** *p*< 0.001. Gender^*a*^, Male = 1, female = 2. Bootstrap sample size = 5000. PMU, problematic mobile phone use; LL, low limit; CI, confidence interval; UL, upper limit. ^*b*^ Empirical 95% confidence interval does not overlap with zero.

**TABLE 5:** Bootstrapping the conditional indirect effect and 95% confidence interval (CI) for the moderated mediation model.

**Indirect path (Alexithymia → acting with awareness → PMU)**		**Estimated effect**	**BootLLCI**	**BootULCI**
**%**	**Moderator: observing (mean centered)**			
16^th^	−5.371	0.019^ a^	0.142	0.246
50^th^	−0.371	0.216^ a^	0.167	0.269
84^th^	4.629	0.242^ a^	0.186	0.300

**N* = 901. Bootstrap sample size = 5000. PMU, problematic mobile phone use; LL, low limit; CI, confidence interval; UL, upper limit. ^*a*^Empirical 95% confidence interval does not overlap with zero.*

## Discussion

Our investigation focused on alexithymia, problematic mobile phone use, and mindfulness facets. By constructing a moderated mediation model, we found that alexithymia positively predicted problematic mobile phones both directly and indirectly via the mindfulness facet of acting with awareness. Acting with awareness mediated this association in a negative way. In addition, the facet of observing moderated the impact of alexithymia on acting with awareness.

### Correlations of the Investigated Variables

It has been determined that alexithymia was negatively related to all the mindfulness facets except observing and non-reactivity to inner experience. This finding contradicts previous studies (de Bruin et al., 2012; MacDonald and Price, 2017). This could be explained by that the observing items of FFMQ largely focus on the awareness of bodily sensations such as the feelings of wind and aroma. This kind of awareness is more typical of mindfulness, cultivated through formal meditation practice, rather than the mindfulness facet that we measure in this study (Owen et al., 2018). Similarly, the items of non-reactivity to inner experience that focus on equanimity and being non-reacting to distressing emotions could also be a quality trained through mindfulness practice rather than a mindfulness facet (Owen et al., 2018).

### Relationship Between Alexithymia and Problematic Mobile Phone Use

In concurrence with previous findings (Gao et al., 2018; Mei et al., 2018; Hao et al., 2019), the results of our investigation showed that alexithymia positively predicted problematic mobile phone use. Individuals with alexithymia are more likely to experience hardships in their lives, which gives them more negative emotions and lower levels of life satisfaction (Mattila et al., 2010; Nekouei et al., 2014). This could be because of the deficit skills in emotion regulation (Ghorbani et al., 2017) and self-control (Fukunishi, 1991). The individuals with alexithymia would choose to escape from the realities and turn to the virtual world provided by mobile phones. If not properly restrained, the dependency on mobile phones would develop into problematic mobile phone use.

### Mediation Effect of Mindfulness Facet

It is indicated that the facet of acting with awareness negatively mediated the impact of alexithymia on the problematic mobile phone, which partially supported our hypotheses. Acting with awareness opposing acting on automatic pilot refers to bringing awareness to the current experiences (Baer et al., 2006). The individuals with alexithymia are less aware of their conditions due to their inabilities to identify and describe the emotions (Parker et al., 1993; Jonason and Krause, 2013). Moreover, these individuals could not stay concentrated on the present experiences (Terry and Terry, 2015; MacDonald and Olsen, 2019). The individuals with alexithymia would gain more awareness of their *status quo*, act with self-consciousness, and get through the conditions when they are trained to have a higher level of acting with awareness. This change would decrease their likelihood of being distracted by problematic behavior such as excessive mobile phone use (Fernandez et al., 2010). Besides, a higher level of acting with awareness brings about more awareness of impulsive actions such as virtual activities, including checking WeChat^1^ Moments^2^ and responding to a post. This helps to refrain from actions that could intrigue problematic mobile phone use (Owen et al., 2018).

### Moderated Mediation Effects of Mindfulness Facets

Our findings supported the hypothesized moderated mediation model between alexithymia and problematic mobile phone use. However, in contrast to our expectations, our results indicated that with higher observing levels, the relationship between alexithymia and acting with awareness has an increasing negative trend. The items of observing have a different meaning for meditators and non-meditators (Baer et al., 2006; de Bruin et al., 2012). Our student sample did not have meditating experiences. High observing level is positively linked with thought suppression for non-meditators (de Bruin et al., 2012). This means that the more one attends to the internal and external experiences, the more likely one deliberately attempts not to think about the negative thoughts. This is in concurrence with previous findings that the self-focused attention may be maladaptive in non-meditators (Mor and Winquist, 2002; Bögels and Mansell, 2004). A high level of attention to feelings (high level of observing) together with a low ability to identify feelings (alexithymia) leads to a constant but unsuccessful effort to identify and understand feelings. This condition results in the change in behavior that the individual does not focus on the present moment and on what he/she is doing but is too much preoccupied with his/her inner life (low acting with awareness).

### Limitations and Implications

Some limitations still need to be addressed in future research. First, the causality between alexithymia and problematic mobile phone use could not be built on the current cross-sectional study. Future longitudinal approaches will be needed to explore in-depth results. Second, the study only included Chinese undergraduate students. The results can be generalizable only to the age group of 18–24 years and were not represented as well for the whole population. Third, the Cronbach’s alpha of the facet observing in this study was somewhat lower than standard, which might affect the analyses and the results.

Despite the limitations, the findings from the current study have important practical implications. This study verified that the mindfulness facet of acting with awareness could effectively attenuate the adverse impact of alexithymia on problematic mobile phone use. Therefore, it is suggested to the college authorities that the problem of the uncontrollable mobile phone uses among students can be addressed by the implementation of training to enhance the level of acting with awareness. Besides, other studies have pointed out that the facet of acting with awareness is a positive construct and is linked with psychological well-being (Bohlmeijer et al., 2011; Calvete et al., 2017).

Another factor that requires our attention is the facet of observing. As suggested by our study, a higher level of observing would enhance the indirect impact of alexithymia on problematic mobile phone use. Individuals who have no experience of meditation will observe the stimuli in a judgmental way (Baer et al., 2006, 2008). Therefore, for students who have high observing levels, training could be offered to foster their non-judgmental attitudes to the negative emotions. In addition, the results of our study showed that high acting with awareness level was significantly related to the low level of observing.

## Conclusion

The current research involved 901 Chinese undergraduate students and examined the moderated mediation effects by mindfulness facets in the association between alexithymia and problematic mobile phone use. While adding evidence of the positive impact of alexithymia on problematic mobile phone use, the study found the mediation role of acting with awareness and the moderation role of observing. Based on our findings, it can be suggested that the education authorities should focus on the specific effects of the mindfulness facets to help the young mobile phone users to stay away from problematic mobile uses.

## Data Availability Statement

The datasets generated for this study are available on request to the corresponding author.

## Ethics Statement

The studies involving human participants were reviewed and approved by Liaoning National Normal College. The patients/participants provided their written informed consent to participate in this study.

## Author Contributions

ZH conceptualized the whole study and was responsible for data collection and analyses and drafted the manuscript. LJ helped the data collection and data analyses. Both authors contributed to the article and approved the submitted version.

## Conflict of Interest

The authors declare that the research was conducted in the absence of any commercial or financial relationships that could be construed as a potential conflict of interest.

## Footnotes

1 WeChat is a mobile/computer based Chinese multi-purpose messaging, social media and mobile payment app developed by Tencent Holdings Limited.

2 Moments is a fundamental feature of WeChat where a user can post text-based updates, upload up to nine images as well as share videos and articles etc.
